# 2,2′-Sulfonyl­dipyrazine 4-oxide

**DOI:** 10.1107/S1600536812028607

**Published:** 2012-06-30

**Authors:** Ai-Min Li, Ya Zhang, Zhi-Wei Wang, Chong-Qing Wan

**Affiliations:** a Department of Chemistry, Capital Normal University, Beijing 100048, People’s Republic of China

## Abstract

In the title compound, C_8_H_6_N_4_O_3_S, the dihedral angle between the pyrazine rings is 85.04 (1)°. In the crystal, mol­ecules are arranged along the *a* axis and are linked by C—H⋯N hydrogen bonds and pyrazine–pyrazine π–π inter­actions [centroid–centroid distance = 3.800 (1) Å, forming an infinite chain array. The chains are connected by C—H⋯O(oxide) hydrogen bonds into layers lying parallel to the *ab* plane. Along the *c* axis, the layers are stacked and linked through C—H⋯O(sulfon­yl) inter­actions, forming a three-dimensional network.

## Related literature

For metal complexes with 2,2′-sulfonyl­dipyrazine, see: Wan & Mak (2011 ▶). For crystal structures of pyridyl-based *N*-oxide and their metal complexes, see: Jia *et al.* (2008 ▶).
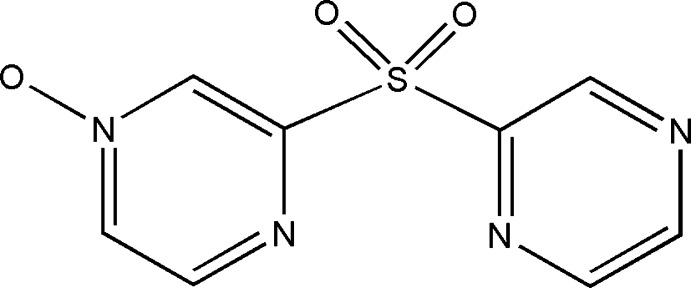


## Experimental

### 

#### Crystal data

C_8_H_6_N_4_O_3_S
*M* *_r_* = 238.23
Monoclinic, 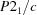

*a* = 7.6860 (16) Å
*b* = 15.841 (3) Å
*c* = 9.0624 (14) Å
β = 117.813 (13)°
*V* = 975.9 (3) Å^3^
*Z* = 4
Mo *K*α radiation
μ = 0.33 mm^−1^
*T* = 296 K
0.45 × 0.30 × 0.25 mm

#### Data collection

Bruker APEXII CCD area-detector diffractometer
Absorption correction: multi-scan (*SADABS*; Bruker, 2007 ▶) *T*_min_ = 0.688, *T*_max_ = 1.000
6606 measured reflections
2429 independent reflections
1586 reflections with *I* > 2σ(*I*)
*R*_int_ = 0.058

#### Refinement

*R*[*F* ^2^ > 2σ(*F* ^2^)] = 0.063
*wR*(*F* ^2^) = 0.203
*S* = 1.07
2429 reflections
145 parameters
H-atom parameters constrained
Δρ_max_ = 0.85 e Å^−3^
Δρ_min_ = −0.46 e Å^−3^

### 

Data collection: *APEX2* (Bruker, 2007 ▶); cell refinement: *APEX2* and *SAINT* (Bruker, 2007 ▶); data reduction: *SAINT*; program(s) used to solve structure: *SHELXS97* (Sheldrick, 2008 ▶); program(s) used to refine structure: *SHELXL97* (Sheldrick, 2008 ▶); molecular graphics: *SHELXTL* (Sheldrick, 2008 ▶); software used to prepare material for publication: *SHELXTL* and *PLATON* (Spek, 2009 ▶).

## Supplementary Material

Crystal structure: contains datablock(s) I, global. DOI: 10.1107/S1600536812028607/zq2171sup1.cif

Structure factors: contains datablock(s) I. DOI: 10.1107/S1600536812028607/zq2171Isup2.hkl

Additional supplementary materials:  crystallographic information; 3D view; checkCIF report

## Figures and Tables

**Table 1:** Hydrogen-bond geometry (Å, °)

*D*—H⋯*A*	*D*—H	H⋯*A*	*D*⋯*A*	*D*—H⋯*A*
C2—H2*A*⋯O1^i^	0.93	2.32	3.130 (5)	146
C3—H3*A*⋯O3^ii^	0.93	2.56	3.419 (4)	153
C7—H7*A*⋯N1^iii^	0.93	2.57	3.449 (3)	157

Symmetry codes: (i) 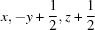; (ii) 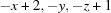; (iii) 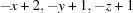.

**Fig. 1.**
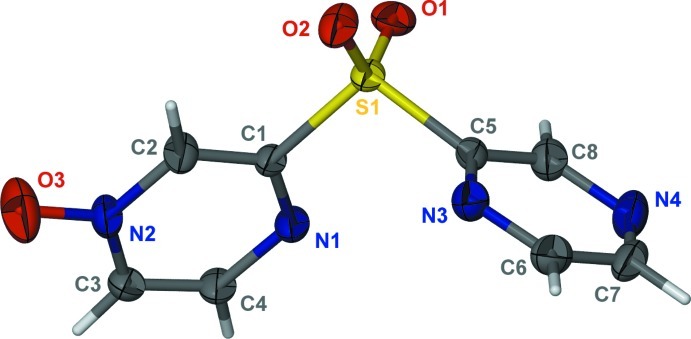
The atom-numbering scheme of the title complex. Displacement ellipsoids are drawn at the 35% probability level and H atoms are shown as sticks of arbitrary radii.

**Fig. 2.**
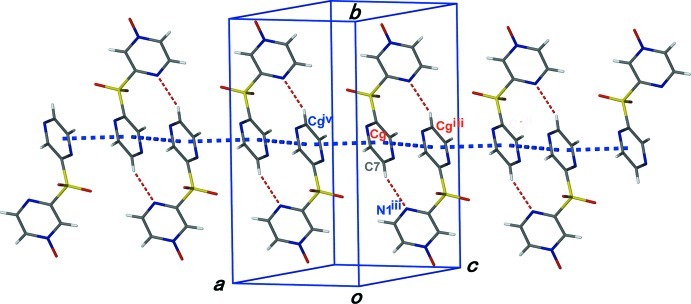
The hydrogen-bonding (C—H···N) and π···π stacking interactions between parallel chains along the a axis, which are respectively shown as thin red-dashed lines and thick blue-dashed lines (symmetry codes: i = - x + 2, - y + 1, - z + 1; ii = x + 1, y, z).

**Fig. 3.**
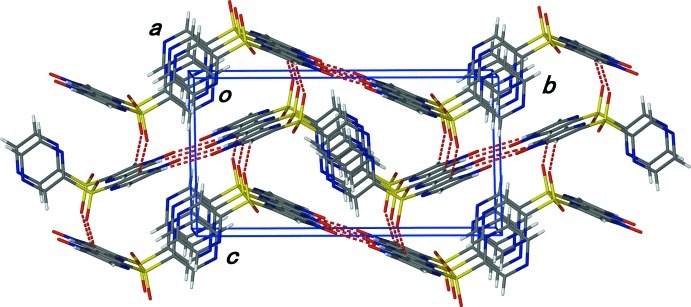
Three-dimensional structure of the title molecule viewed down the a direction. The red dashed lines represent hydrogen-bonding interactions.

## supplementary crystallographic information

### Comment

Pyridyl based sulfonyl derivatives were widely used in supramolecular assemblies of transition metal complexes (Wan & Mak, 2011). Pyridyl based N-oxide derivatives have also been demonstrated as versatile building blocks to construct supramolecular architectures of various metal complexes (Jia *et al.*, 2008). In the present context, we report the structure of the title compound, a new N-oxide compound derived from 2,2'-sulfonyldipyrazine.

In the title compound, the value of the C1(sp^2^)—S1—C5(sp^2^) angle is 103.92 (1)° with two attached pyrazinyl rings exhibiting a dihedral angle of 85.04 (1)°, as shown in Fig. 1. The angular-shaped molecules are arranged along the *a* axis. As shown in Fig. 2, two adjacent molecules arranged with an inversion center are interconnected through C7—H7A···N1*^iii^* and π···π interactions (Cg···Cg*^iii^* = 3.800 (1) Å, Cg represents the C5-N3-C6-C7-N4-C8 ring; symmetry code: *iii* = 2 - *x*, 1 - *y*, 1 - *z*). The dimers are further interconnected through π···π interactions between Cg and Cg*^iv^* [Cg···Cg*^iv^* = 4.174 (2) Å, the distance between the closest ring atom and one Cg is 3.597 (2) Å; symmetry code = 1 - *x*, 1 - *y*, 1 - *z*]. The formed chains are further connected through C3—H3A···O3*^ii^*(oxynitride) hydrogen bonds to form a layer almost parallel to the *ab* plane (symmetry code: *ii* = 2 - *x*, - *y*, 1 - *z*). Along the *c* axis, the formed layers are stacked and interconnected through C2—H2A···O1*^i^*(sulfonyl) interactions to form a three-dimensional framework (Fig. 3, Table 1; symmetry code: *i* = *x*, -*y* + 1/2, *z* + 1/2).

### Experimental

The title compound was obtained as a serendipitous byproduct as the 2,2'-dipyrazine sulfide (0.022 g, 0.1 mmol) was dissolved in a mixture of methanol 2 ml and acetonitrile 2 ml to react with Mn(ClO_4_)_2_.6H_2_O (0.036 g, 0.1 mmol) with constantly stirring at room temperature. After three hours, the clear solution was filtrated and kept in air for about one week to yield colourless block crystals (7 mg, 29% yield). We got the the title compound as a matter of the oxidability by perchlorate acid from Mn(ClO_4_)_2_.6H_2_O.

### Refinement

All hydrogen positions were calculated after each cycle of refinement using a riding model with C—H = 0.93 Å and *U*_iso_(H) = 1.2*U*_eq_(C). The highest peak (0.8 e.Å^-3^) in the difference Fourier map is located at 1.1 Å from atom N4. The refinement of a model including one H atom at this position led to lower R_1_ and wR_2_ values but it is chemically meaningless since there is no counter ion in the crystal structure. A positional disorder of the oxo O atom (partially on atoms O4 and O2) is surely the best solution but in this case too many restraints had to be used in the final refinements to get an acceptable model (with an site-occupancy ratio greater than 0.9:0.1).

### Crystal data

**Table:**

C_8_H_6_N_4_O_3_S	*F*(000) = 488
*M**_r_* = 238.23	*D*_x_ = 1.621 Mg m^−^^3^*D*_m_ = 1.621 Mg m^−^^3^*D*_m_ measured by not measured
Monoclinic, *P*2_1_/*c*	Mo *K*α radiation, λ = 0.71073 Å
Hall symbol: -P 2ybc	Cell parameters from 365 reflections
*a* = 7.6860 (16) Å	θ = 2.6–28.4°
*b* = 15.841 (3) Å	µ = 0.33 mm^−^^1^
*c* = 9.0624 (14) Å	*T* = 296 K
β = 117.813 (13)°	Needle-like, colourless
*V* = 975.9 (3) Å^3^	0.45 × 0.30 × 0.25 mm
*Z* = 4	

### Data collection

**Table:**

Bruker APEXII CCD area-detector diffractometer	2429 independent reflections
Radiation source: fine-focus sealed tube	1586 reflections with *I* > 2σ(*I*)
Graphite monochromator	*R*_int_ = 0.058
ω scans	θ_max_ = 28.4°, θ_min_ = 2.6°
Absorption correction: multi-scan (*SADABS*; Bruker, 2007)	*h* = −10→10
*T*_min_ = 0.688, *T*_max_ = 1.000	*k* = −21→11
6606 measured reflections	*l* = −12→11

### Refinement

**Table:**

Refinement on *F*^2^	Primary atom site location: structure-invariant direct methods
Least-squares matrix: full	Secondary atom site location: difference Fourier map
*R*[*F*^2^ > 2σ(*F*^2^)] = 0.063	Hydrogen site location: inferred from neighbouring sites
*wR*(*F*^2^) = 0.203	H-atom parameters constrained
*S* = 1.07	*w* = 1/[σ^2^(*F*_o_^2^) + (0.0981*P*)^2^ + 0.5688*P*] where *P* = (*F*_o_^2^ + 2*F*_c_^2^)/3
2429 reflections	(Δ/σ)_max_ < 0.001
145 parameters	Δρ_max_ = 0.85 e Å^−^^3^
0 restraints	Δρ_min_ = −0.46 e Å^−^^3^

### Special details

**Table:**

Geometry. All esds (except the esd in the dihedral angle between two l.s. planes) are estimated using the full covariance matrix. The cell esds are taken into account individually in the estimation of esds in distances, angles and torsion angles; correlations between esds in cell parameters are only used when they are defined by crystal symmetry. An approximate (isotropic) treatment of cell esds is used for estimating esds involving l.s. planes.
Refinement. Refinement of F^2^ against ALL reflections. The weighted R-factor wR and goodness of fit S are based on F^2^, conventional R-factors R are based on F, with F set to zero for negative F^2^. The threshold expression of F^2^ > 2sigma(F^2^) is used only for calculating R-factors(gt) etc. and is not relevant to the choice of reflections for refinement. R-factors based on F^2^ are statistically about twice as large as those based on F, and R- factors based on ALL data will be even larger.

### Fractional atomic coordinates and isotropic or equivalent isotropic displacement parameters (Å^2^)

**Table:**

	*x*	*y*	*z*	*U*_iso_*/*U*_eq_	
S1	0.50844 (11)	0.33579 (5)	0.25247 (12)	0.0436 (3)	
N1	0.8484 (4)	0.27476 (19)	0.2912 (4)	0.0531 (8)	
N2	0.8080 (4)	0.12101 (18)	0.4199 (4)	0.0520 (8)	
N3	0.6907 (4)	0.4321 (2)	0.5196 (4)	0.0540 (8)	
N4	0.7631 (6)	0.5635 (2)	0.3499 (6)	0.0699 (10)	
O1	0.4354 (3)	0.35188 (18)	0.0788 (3)	0.0581 (7)	
O2	0.3755 (4)	0.31172 (17)	0.3141 (4)	0.0627 (8)	
O3	0.7852 (6)	0.0494 (2)	0.4742 (6)	0.0996 (13)	
C1	0.6949 (4)	0.25624 (19)	0.3152 (4)	0.0403 (7)	
C2	0.6667 (5)	0.1820 (2)	0.3776 (5)	0.0494 (9)	
H2A	0.5552	0.1732	0.3910	0.059*	
C3	0.9681 (5)	0.1382 (2)	0.4026 (5)	0.0535 (9)	
H3A	1.0685	0.0985	0.4351	0.064*	
C4	0.9844 (5)	0.2134 (2)	0.3375 (6)	0.0582 (10)	
H4A	1.0959	0.2229	0.3244	0.070*	
C5	0.6391 (4)	0.4281 (2)	0.3598 (4)	0.0416 (8)	
C6	0.7790 (6)	0.5037 (3)	0.5944 (6)	0.0627 (11)	
H6A	0.8189	0.5105	0.7076	0.075*	
C7	0.8125 (6)	0.5678 (3)	0.5086 (7)	0.0677 (13)	
H7A	0.8734	0.6167	0.5664	0.081*	
C8	0.6737 (5)	0.4914 (2)	0.2717 (5)	0.0561 (9)	
H8A	0.6355	0.4843	0.1588	0.067*	

### Atomic displacement parameters (Å^2^)

**Table:**

	*U*^11^	*U*^22^	*U*^33^	*U*^12^	*U*^13^	*U*^23^
S1	0.0286 (4)	0.0384 (4)	0.0661 (6)	−0.0005 (3)	0.0238 (4)	−0.0064 (4)
N1	0.0336 (13)	0.0392 (15)	0.090 (2)	−0.0016 (11)	0.0314 (14)	0.0025 (14)
N2	0.0566 (17)	0.0332 (14)	0.073 (2)	0.0047 (13)	0.0357 (16)	0.0039 (14)
N3	0.0531 (17)	0.0451 (17)	0.062 (2)	0.0018 (13)	0.0250 (15)	−0.0052 (14)
N4	0.069 (2)	0.0405 (17)	0.110 (3)	−0.0067 (16)	0.050 (2)	−0.0055 (19)
O1	0.0378 (12)	0.0647 (17)	0.0632 (17)	0.0072 (11)	0.0165 (11)	−0.0084 (13)
O2	0.0493 (14)	0.0492 (15)	0.109 (2)	−0.0070 (12)	0.0529 (15)	−0.0115 (14)
O3	0.117 (3)	0.0558 (19)	0.159 (4)	0.020 (2)	0.092 (3)	0.033 (2)
C1	0.0313 (14)	0.0330 (15)	0.0563 (19)	−0.0009 (12)	0.0203 (13)	−0.0058 (14)
C2	0.0477 (18)	0.0394 (17)	0.074 (2)	0.0008 (14)	0.0395 (18)	−0.0013 (16)
C3	0.0350 (16)	0.0456 (19)	0.072 (2)	0.0044 (14)	0.0187 (16)	−0.0014 (18)
C4	0.0315 (16)	0.0463 (19)	0.099 (3)	0.0001 (14)	0.0318 (18)	0.0007 (19)
C5	0.0311 (14)	0.0333 (15)	0.062 (2)	0.0036 (12)	0.0230 (14)	−0.0017 (14)
C6	0.055 (2)	0.056 (2)	0.068 (3)	0.0036 (18)	0.0203 (19)	−0.017 (2)
C7	0.047 (2)	0.041 (2)	0.115 (4)	−0.0084 (16)	0.038 (2)	−0.024 (2)
C8	0.055 (2)	0.045 (2)	0.072 (3)	0.0007 (16)	0.0323 (19)	−0.0001 (18)

### Geometric parameters (Å, º)

**Table:**

S1—O1	1.425 (3)	N4—C8	1.351 (5)
S1—O2	1.426 (3)	C1—C2	1.366 (5)
S1—C5	1.784 (3)	C2—H2A	0.9300
S1—C1	1.790 (3)	C3—C4	1.362 (6)
N1—C1	1.328 (4)	C3—H3A	0.9300
N1—C4	1.343 (4)	C4—H4A	0.9300
N2—O3	1.281 (4)	C5—C8	1.382 (5)
N2—C3	1.339 (5)	C6—C7	1.374 (7)
N2—C2	1.368 (4)	C6—H6A	0.9300
N3—C5	1.312 (5)	C7—H7A	0.9300
N3—C6	1.333 (5)	C8—H8A	0.9300
N4—C7	1.306 (6)		
			
O1—S1—O2	119.66 (17)	N2—C3—C4	120.2 (3)
O1—S1—C5	106.68 (17)	N2—C3—H3A	119.9
O2—S1—C5	109.23 (16)	C4—C3—H3A	119.9
O1—S1—C1	108.72 (16)	N1—C4—C3	123.6 (3)
O2—S1—C1	107.53 (17)	N1—C4—H4A	118.2
C5—S1—C1	103.92 (14)	C3—C4—H4A	118.2
C1—N1—C4	114.3 (3)	N3—C5—C8	124.2 (3)
O3—N2—C3	121.5 (3)	N3—C5—S1	116.4 (3)
O3—N2—C2	120.0 (3)	C8—C5—S1	119.4 (3)
C3—N2—C2	118.5 (3)	N3—C6—C7	121.7 (4)
C5—N3—C6	114.9 (4)	N3—C6—H6A	119.1
C7—N4—C8	115.9 (4)	C7—C6—H6A	119.1
N1—C1—C2	125.6 (3)	N4—C7—C6	123.4 (4)
N1—C1—S1	115.7 (2)	N4—C7—H7A	118.3
C2—C1—S1	118.6 (2)	C6—C7—H7A	118.3
C1—C2—N2	117.7 (3)	N4—C8—C5	119.8 (4)
C1—C2—H2A	121.1	N4—C8—H8A	120.1
N2—C2—H2A	121.1	C5—C8—H8A	120.1
			
C4—N1—C1—C2	−1.3 (5)	N2—C3—C4—N1	1.6 (7)
C4—N1—C1—S1	−179.2 (3)	C6—N3—C5—C8	1.0 (5)
O1—S1—C1—N1	59.3 (3)	C6—N3—C5—S1	−176.7 (3)
O2—S1—C1—N1	−169.8 (3)	O1—S1—C5—N3	169.1 (2)
C5—S1—C1—N1	−54.1 (3)	O2—S1—C5—N3	38.4 (3)
O1—S1—C1—C2	−118.8 (3)	C1—S1—C5—N3	−76.1 (3)
O2—S1—C1—C2	12.1 (3)	O1—S1—C5—C8	−8.7 (3)
C5—S1—C1—C2	127.8 (3)	O2—S1—C5—C8	−139.4 (3)
N1—C1—C2—N2	0.1 (6)	C1—S1—C5—C8	106.1 (3)
S1—C1—C2—N2	178.0 (3)	C5—N3—C6—C7	−0.1 (5)
O3—N2—C2—C1	−177.9 (4)	C8—N4—C7—C6	0.5 (6)
C3—N2—C2—C1	1.9 (5)	N3—C6—C7—N4	−0.6 (6)
O3—N2—C3—C4	177.1 (4)	C7—N4—C8—C5	0.3 (6)
C2—N2—C3—C4	−2.8 (6)	N3—C5—C8—N4	−1.1 (5)
C1—N1—C4—C3	0.4 (6)	S1—C5—C8—N4	176.5 (3)

### Hydrogen-bond geometry (Å, º)

**Table:**

*D*—H···*A*	*D*—H	H···*A*	*D*···*A*	*D*—H···*A*
C2—H2*A*···O1^i^	0.93	2.32	3.130 (5)	146
C3—H3*A*···O3^ii^	0.93	2.56	3.419 (4)	153
C7—H7*A*···N1^iii^	0.93	2.57	3.449 (3)	157

Symmetry codes: (i) *x*, −*y*+1/2, *z*+1/2; (ii) −*x*+2, −*y*, −*z*+1; (iii) −*x*+2, −*y*+1, −*z*+1.
